# Striatal Dopamine Depletion Patterns and Early Non-Motor Burden in Parkinsons Disease

**DOI:** 10.1371/journal.pone.0161316

**Published:** 2016-08-16

**Authors:** Su Jin Chung, Jae Jung Lee, Jee Hyun Ham, Byoung Seok Ye, Phil Hyu Lee, Young H. Sohn

**Affiliations:** 1 Department of Neurology, Yonsei University College of Medicine, Seoul, South Korea; 2 Department of Neurology, Inje University Ilsan Paik Hospital, Goyang, South Korea; 3 Severance Biomedical Science Institute, Yonsei University College of Medicine, Seoul, South Korea; Hudson Institute, AUSTRALIA

## Abstract

**Background:**

The mechanism underlying non-motor symptoms in Parkinson’s disease has not yet been elucidated. In this study, we hypothesized that Parkinson patients with more non-motor symptoms have a different pattern of striatal dopamine depletion, particularly in areas other than the sensorimotor striatum, compared to those with fewer non-motor symptoms.

**Methods:**

We conducted a prospective survey of the degree of non-motor symptoms (using the Korean version of the Non-Motor Symptoms Scale; K-NMSS) in 151 patients with early-stage Parkinson’s disease who had undergone a dopamine transporter PET scan as an initial diagnostic procedure. We classified the patients into two groups; high non-motor patients (HNM-PD; K-NMSS score ≥ 41) and low non-motor patients (LNM-PD).

**Results:**

Patients in the HNM-PD group (*n* = 71) were older, had longer symptom duration, exhibited more severe motor deficits, and had been prescribed higher levodopa-equivalent doses at follow-up than those in the LNM-PD group. However, dopamine transporter binding to the striatal sub-regions and inter-sub-regional binding ratios were comparable between the two groups. A general linear model showed that the HNM-PD group had significantly more severe motor deficits than the LNM-PD group after controlling for age, gender, symptom duration, and dopamine transporter binding to the sensorimotor striatum.

**Conclusions:**

This study demonstrated that the pattern of striatal dopamine depletion does not contribute to early non-motor burden in Parkinson’s disease. Our results suggest that LNM-PD patients may have a more benign course of motor symptom progression than HNM-PD patients.

## Introduction

Parkinson’s disease (PD) is accompanied by a variety of non-motor symptoms (NMSs) involving sleep, mood, cognition, attention, and autonomic functions [1]. NMSs can occur across all stages of PD, and have been recognized as a key determinant factor for quality of life in PD patients [2]. The motor symptoms of PD result mainly from the loss of dopaminergic neurons in the nigrostriatal pathway; however, the neuroanatomical and neurochemical substrates of NMSs in PD remain unknown [1]. Furthermore, PD pathology also involves widespread non-dopaminergic brainstem and cortical lesions, even before the emergence of motor symptoms [3,4]. Due to the correlation between the premotor occurrence of NMSs and the progression of PD pathology in non-dopaminergic systems, non-dopaminergic system involvement has been proposed to be a key mechanism responsible for NMSs in PD [5,6]. However, as certain NMSs and non-motor fluctuations are improved via dopaminergic treatment as well as deep brain stimulation, a dopaminergic pathophysiology of NMSs, particularly involving brain areas other than the nigrostriatal system, has also been suggested [2].

The basal ganglia are organized into sensorimotor, associative, and limbic regions [7]. While the motor and premotor cortex projecting to the posterior and ventral putamen act to control sensorimotor function (the sensorimotor striatum), the head of the caudate nucleus and the anterior putamen, receiving inputs from the dorsolateral prefrontal cortex, are involved in working memory (the associative striatum), and the ventral striatum, linking to the orbital and medial prefrontal cortex, is in charge of primary rewards with motivation and emotion (the limbic striatum) [8]. In PD patients, dopamine transporter (DAT) activity is significantly reduced in all of these sub-regions of the striatum, although the degree of reduction differs among the sub-regions [9,10]. Furthermore, inter-sub-regional ratios also differ among various forms of Parkinsonism, suggesting that the diverse patterns of striatal dopaminergic depletion contribute to different clinical phenotypes in degenerative parkinsonism [10]. Therefore, we performed this study to investigate primarily whether the pattern of striatal dopamine depletion contributes to the early burden of NMSs in PD. We hypothesized that PD patients with a greater burden of NMSs might have a different pattern of striatal dopamine depletion from those with fewer NMSs (i.e., greater depletion in either the limbic or the associative striatum). In addition, a recent cluster analysis study demonstrated that non-motor dominant patients with PD showed a faster motor progression than either pure-motor or mixed (motor/non-motor) patients [11]. Thus, secondly, we also investigated whether a greater burden of NMSs influenced motor deficits in relation to dopamine depletion in the sensorimotor striatum.

## Materials and Methods

### Patients

We performed a survey assessing the degree of NMSs, using the Korean version of the Non-Motor Symptoms Scale (K-NMSS) [12], in patients with early-stage PD who had been initially diagnosed at Yonsei Parkinson Center via DAT scan, using an [^18^F] N-(3-Fluoropropyl)-2β-carbon ethoxy-3β-(4-iodophenyl) nortropane (FP-CIT) PET scan. PD in these patients was diagnosed according to the clinical criteria of the UK PD Brain Bank [13], the presence of appropriate DAT defects on the FP-CIT PET scans [10], and the presence of a PD drug response for longer than 15 months during the follow-up period. The interpretation of the FP-CIT-PET scans was performed by nuclear medicine physicians who were blind to the clinical status of each patient. Part III of the Unified Parkinson’s Disease Rating Scale (UPDRS-motor) was used to estimate PD motor severity in a drug-naive state at the time of FP-CIT PET acquisition. The Mini-Mental State Examination (MMSE) was performed as a routine diagnostic procedure. Parkinsonian medications prescribed at the time of K-NMSS assessment were checked. Follow-up duration was calculated as the period between the date of initiation of PD therapy and the date of K-NMSS assessment. Levodopa-equivalent dose was calculated according to a method described in a previous study [14]. We received approval from the human research protection center of Severance hospital for experiments using human participants, and obtained written informed consent from each patient.

### Assessment of non-motor symptoms

The original Non-Motor Symptoms Scale was developed for the assessment of NMSs in PD patients by the International Parkinson’s Disease Non-Motor Group [15] and has been widely used in clinical studies of PD. The K-NMSS has also been developed and validated [12]. It is composed of 30 items grouped into nine domains: cardiovascular including falls (two items), sleep/fatigue (four items), mood/cognition (six items), perceptual problems/ hallucinations (three items), attention/memory (three items), gastrointestinal tract (three items), urinary (three items), sexual function (two items), and miscellaneous (four items; pain, taste or smell, weight change, and excessive sweating). The score for each item is a multiple of scores for severity (from 0 to 3) and frequency (from 1 to 4). A total K-NMSS score ranges from 0 to 360, with a higher score representing more severe NMSs. K-NMSS scores were assigned by a neurologist on the basis of interviews with the patients and their caregivers. We divided the patients into two groups according to total K-NMSS scores: patients who showed a K-NMSS score of 41 or higher were defined as high non-motor PD (HNM-PD), while those with K-NMSS scores of 40 or lower were classified as low non-motor PD (LNM-PD). This classification was adopted by a recent proposal for a comprehensive grading of NMSs severity in PD [16], in which severe and very severe NMS burden corresponded to HNM-PD in this study.

### PET-CT image acquisition and quantitative analysis of PET data

DAT scans using FP-CIT with a GE Discovery STe (DSTE) PET-CT scanner (GE Healthcare Technologies, Milwaukee, WI, USA) were performed to estimate striatal dopamine depletion. The details of PET-CT image acquisition were the same as those have previously described [17].

Quantitative analyses of FP-CIT PET data were conducted based on volumes of interest (VOIs): 12 VOIs of bilateral striatal sub-regions and one occipital VOI were drawn in the same way as described in previous studies [10,17]. Each VOI was anatomically defined according to the criteria described in the previous study [10]. Using the DAT concentration in each VOI, DAT binding to each VOI was assessed as follows: (mean standardized uptake value [SUV] of the striatal sub-region VOI—mean SUV of the occipital VOI) / mean SUV of the occipital VOI. We categorized DAT activity in the striatal sub-regions into three parts: limbic (DAT activity in the ventral striatum), associative (sum of DAT activity in the anterior and posterior caudate and in the anterior putamen), and sensorimotor (sum of DAT activity in the ventral and posterior putamen). This method was based on previous neuroanatomical studies [7,8].

### Statistical analysis

Data are expressed as means ± standard deviations. An independent *t* test was used to compare numeric variables, and a χ^2^ test was used to compare categorical variables between the HNM-PD and LNM-PD group. A Pearson correlation analysis was performed to analyze the correlation between initial motor deficits and DAT binding to the sensorimotor striatum, as well as between total K-NMSS score and DAT binding to the striatal sub-regions. The predictive value of initial clinical variables for early non-motor burden was analyzed using a multivariate regression analysis. To investigate whether a greater burden of NMSs influences motor deficits in relation to dopamine depletion in the sensorimotor striatum, a general linear model was used to compare the difference in initial UPDRS-motor scores between the two groups after controlling for potential confounding factors, such as age, gender and symptom duration. A general linear model was also employed to compare the difference in levodopa-equivalent doses at follow-up between the two groups after controlling for follow-up duration. SPSS Statistics 21 (IBM SPSS, Armonk, NY, USA) was used to perform all statistical analyses. *P*–values of <0.05 were regarded as significant.

## Results

A total of 151 patients (mean age, 67.9 ± 9.6 years; range, 41–88 years; 67 men) were included in this study. The follow-up duration ranged from 16 to 67 months (mean, 40.3 ± 15.1 months). The total K-NMSS score for each patient ranged from 0 to 169 (mean, 45.5 ± 34.0; median, 37). Seventy-one patients (47.0%) belonged to the HNM-PD group. The clinical characteristics and striatal DAT binding of the two groups are shown in Table 1. The HNM-PD group was older, had longer symptom duration, exhibited higher initial UPDRS-motor scores, and was prescribed higher levodopa-equivalent doses at follow-up than the LNM-PD group. Other variables including DAT binding to the striatal sub-regions and inter-sub-regional ratios were comparable between the two groups. Initial UPDRS-motor score was negatively correlated with DAT binding to the sensorimotor striatum (*r* = -0.314, *p* < 0.001; S1 Fig). However, total K-NMSS score was not correlated with DAT binding to the striatal sub-regions (S2 Fig).

**Table 1:** Clinical characteristics and dopamine transporter binding to the striatal sub-regions.

	LMN-PD (*n* = 80)	HNM-PD (*n* = 71)	*p*-value
**Age (years)**	65.9 ± 10.1	70.2 ± 8.4	0.006
**Gender (% man)**	46.2	42.3	0.627
**Symptom duration (months)**^a^	52.5 ± 19.5	61.3 ± 21.0	0.009
**Follow-up duration (months)**^b^	38.4 ± 15.1	42.3 ± 15.0	0.115
**Initial UPDRS-motor score**^c^	20.8 ± 10.6	27.4 ± 11.4	0.001
**MMSE score**^c^	25.9 ± 2.8	25.6 ± 3.7	0.495
**Levodopa-equivalent dose (mg)**	517 ± 197	631 ± 240	0.002
**DAT binding to striatal sub-regions**^c^			
Limbic	2.40 ± 0.58	2.29 ± 0.65	0.279
Associative	5.58 ± 1.78	5.41 ± 1.86	0.583
Sensorimotor	2.62 ± 0.74	2.51 ± 0.81	0.406
**Inter-sub-regional DAT binding ratios**^c^			
Limbic/Sensorimotor	0.95 ± 0.23	0.95 ± 0.25	0.972
Associative/Sensorimotor	2.14 ± 0.39	2.18 ± 0.43	0.637

Data are means ± standard deviations.

LMN-PD, low non-motor Parkinson’s disease; HNM-PD, high non-motor Parkinson’s disease; UPDRS, Unified Parkinson’s Disease Rating Scale; MMSE, Mini-Mental State Examination; DAT, dopamine transporter.

^a^ Duration from symptom onset to K-NMSS assessment

^b^ Duration from medication start to K-NMSS assessment

^c^ Measures perfomed at baseline

To evaluate the value of initial clinical variables and striatal DAT binding predicting early non-motor burden in PD, a multivariate logistic regression analysis was performed with HNM-PD as the dependent variable, and patient age, symptom duration, initial UPDRS-motor score, and DAT binding to the striatal sub-regions as the independent variables. Given that the striatal sub-regional DAT bindings were significantly correlated to each other (*r* > 0.7), each sub-regional DAT binding was applied in separately multivariate logistic regression analyses. This analysis revealed that symptom duration, initial motor deficits and patient age, though not DAT binding to the striatal sub-regions, were important factors for predicting early non-motor burden in our patients (Table 2).

**Table 2:** Independent predictors of early non-motor burden in Parkinson’s disease.

	*β*	OR (95% CI)	*p*-value
**Limbic striatum**			
Age (years)	0.054	1.055 (1.006–1.107)	0.027
Symptom duration (months)	0.037	1.038 (1.016–1.107)	0.001
UPDRS motor score	0.059	1.060 (1.020–1.102)	0.003
DAT binding	0.110	1.116 (0.575–2.167)	0.745
**Associative striatum**			
Age (years)	0.058	1.060 (1.008–1.114)	0.024
Symptom duration (months)	0.037	1.037 (1.016–1.060)	0.001
UPDRS motor score	0.059	1.061 (1.021–1.103)	0.003
DAT binding	0.075	1.078 (0.850–1.368)	0.534
**Sensorimotor striatum**			
Age (years)	0.050	1.051 (1.004–1.100)	0.032
Symptom duration (months)	0.037	1.037 (1.016–1.060)	0.001
UPDRS motor score	0.056	1.058 (1.017–1.100)	0.005
DAT binding	-0.090	0.914 (0.526–1.588)	0.749

*β*, logistic regression coefficient; OR, odds ratio; CI, confidence interval; UPDRS, Unified Parkinson’s Disease Rating Scale; DAT, dopamine transporter.

**Fig 1:**
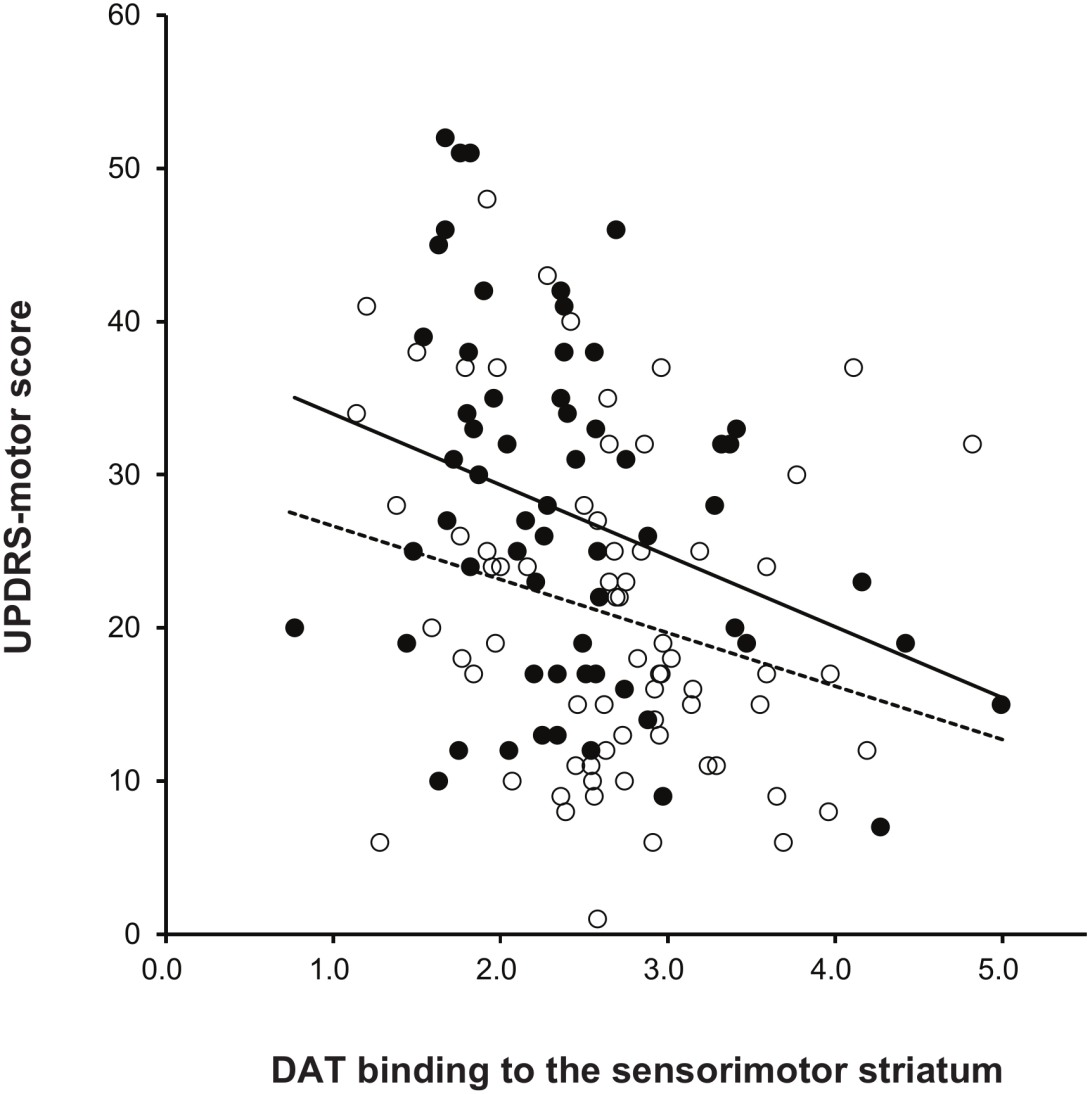
UPDRS-motor score and dopamine transporter (DAT) binding between the two groups. HNM-PD (closed circle) showed higher UPDRS-motor scores than LNM-PD (open circle) at similar DAT level. However, group (HNM-PD, solid line/LNM-PD, dotted line) and DAT activity interaction was non-significant for UPDRS-motor scores.

**Fig 2:**
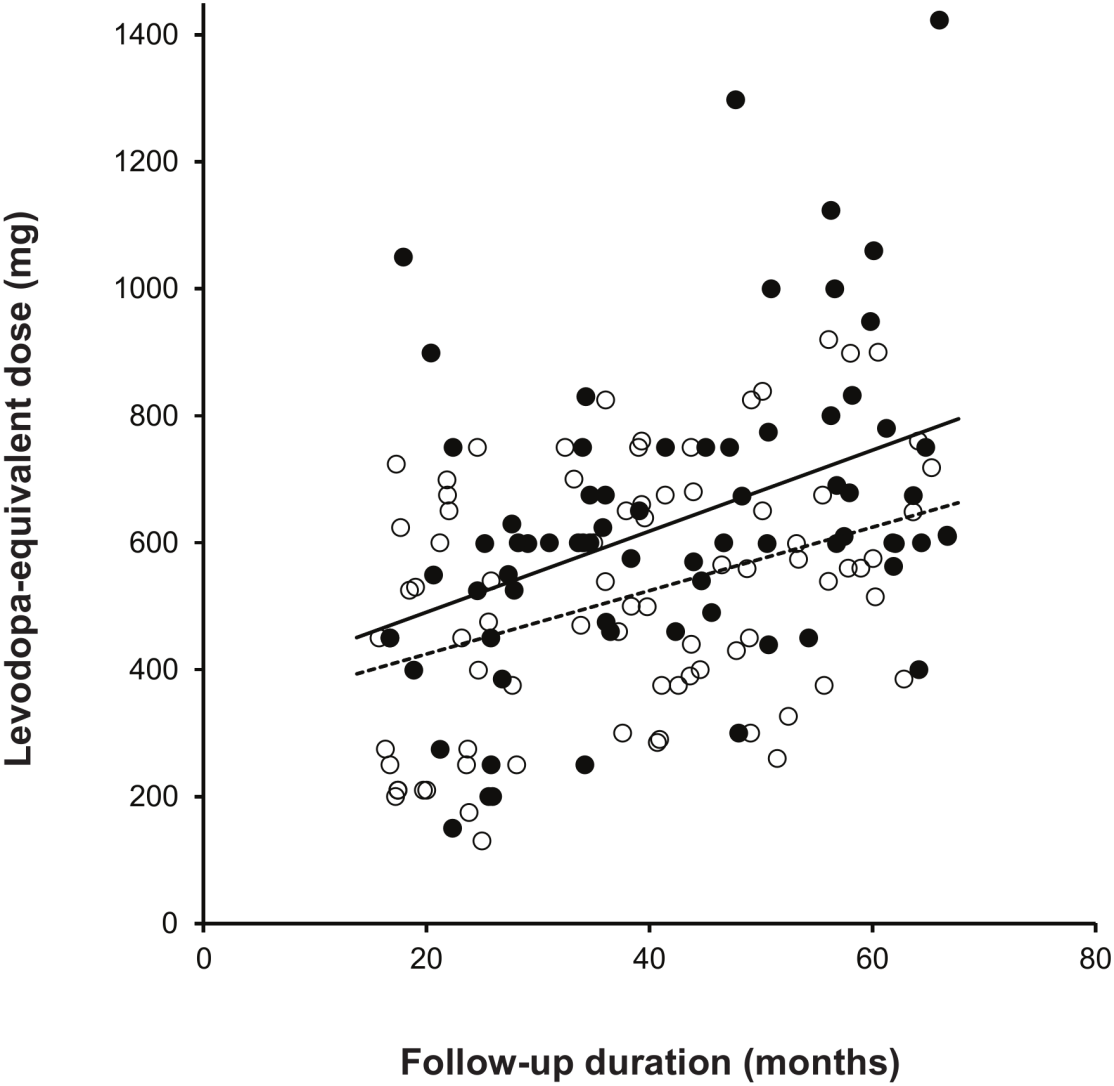
Levodopa-equivalent dose and follow-up duration between the two groups. HNM-PD (closed circle) received higher levodopa-equivalent dose than LNM-PD (open circle) at similar follow-up duration. However, group (HNM-PD, solid line/LNM-PD, dotted line) and follow-up duration interaction was non-significant for levodopa-equivalent doses.

A general linear model showed that the HNM-PD group exhibited significantly higher initial UPDRS-motor scores than the LNM-PD group after controlling for age, gender, symptom duration, and DAT binding to the sensorimotor striatum (*p* = 0.004; Table 3, Fig 1). However, the effect of the interaction between patient group (HNM-PD/LNM-PD) and DAT binding to the sensorimotor striatum on UPDRS-motor scores was not significant (*p* = 0.555). A general linear model also showed that a levodopa-equivalent dose at follow-up was higher in the HNM-PD group than in the LNM-PD group after controlling for follow-up duration (*p* = 0.005; Fig 2). However, the effect of the interaction between patient group (HNM-PD/LNM-PD) and follow-up duration on levodopa-equivalent doses was not significant (*p* = 0.532).

**Table 3:** Effect of early non-motor burden and dopamine transporter binding to the sensorimotor striatum on initial UPDRS-motor score.

	Unadjusted		Adjusted^a^	
	*β* (S.E.)	*p*-value	*β* (S.E)	*p*-value
**DAT binding**	-4.05 (1.22)	0.001	-3.78 (1.27)	0.004
**Early non-motor burden**				
LNM-PD	-5.54 (1.88)	0.004	-5.90 (1.99)	0.004
HNM-PD	Reference		Reference	

*β*, estimated slope; S.E., standard error; DAT, dopamine transporter; LMN-PD, low non-motor Parkinson’s disease; HNM-PD, high non-motor Parkinson’s disease.

^a^ Adjusted for age, gender, symptom duration, and DAT binding to the sensorimotor striatum.

## Discussion

The results demonstrate that the HNM-PD group showed different clinical characteristics, such as an older age, longer symptom duration, and more initial motor deficits at baseline, when compared to the LNM-PD group. The mean K-NMSS score in our patients was lower than that in the original study of the Non-Motor Symptoms Scale [15], presumably due to our enrollment of early-stage patients. In the original study, Non-Motor Symptoms Scale score correlated to onset age, duration of PD, and UPDRS score, which is consistent with the present results.

Contrary to our initial hypothesis, DAT binding to the striatal sub-regions and inter-sub-regional ratios were similar between the HNM-PD and LNM-PD groups. Furthermore, a multivariate analysis also revealed that symptom duration, initial motor deficits, and patient age, though not DAT binding to the striatal sub-regions, were important factors for predicting early non-motor burden in our patients. This result suggests that NMSs in PD are not associated with the pattern of striatal dopamine depletion but are more likely related to either non-striatal dopamine depletion or non-dopaminergic lesions in PD. A previous neuroimaging study showing dopamine dysfunction in the hypothalamus of patients with PD suggests a potential contribution of non-striatal dopamine dysfunction to certain NMSs [18]. A recent study showing a negative correlation between the severity of NMSs and resting functional connectivity centered on the inferior orbito-frontal area [19] also supports the contribution of non-striatal lesions to NMSs in PD. In addition, as older age is also an important factor for early non-motor burden, aging-related medical comorbidities may play an important role in the pathogenesis of NMSs in PD. However, certain NMSs might be more under the influence of striatal dopamine than others. Dopaminergic medications may ameliorate or worsen NMSs such as sleep/fatigue, mood, gastrointestinal and urinary symptoms, sexual dysfunction [2]. Thus, using a total K-NMSS score may obscure the possible role of dopamine in these NMSs.

Higher motor scores in the HNM-PD group than in the LNM-PD group remained significant after controlling DAT binding to the sensorimotor striatum as well as age, gender, and motor symptom duration in each patient, suggesting that despite similar dopamine depletion, the LNM-PD group has significantly fewer motor deficits than the HNM-PD group. This result could indicate that the LNM-PD group shows a more benign course or a greater reserve capacity, compensating for the pathological processes, when compared to the HNM-PD group. This finding supports the previous observation that non-motor-dominant PD patients show a faster motor progression than both pure-motor and mixed (motor/non-motor) patients [11]. The fewer levodopa-equivalent doses in the LNM-PD group when compared to the HNM-PD group after similar follow-up duration may also support a more benign course in the LNM-PD group. If the premotor or early occurrence of NMSs is well correlated to the progression of PD pathology in widespread brainstem and cortical regions, the LNM-PD group may represent either less pathological involvement or greater compensatory ability for pathological processes (i.e., fewer NMSs despite similar pathological involvement) in PD. A similar mechanism might act to produce fewer motor deficits in the LNM-PD group than the HNM-PD group, despite similar level of dopamine depletion in the sensorimotor striatum.

In this study, striatal DAT binding and UPDRS-motor score were checked in a drug naive state, which excluded the influence of any PD medication on these measurements. A programmed measure of striatal DAT binding minimized inter- and intra-rater variability, and consecutive patient sampling also minimized patient-sampling bias. Nevertheless, this study had several notable limitations. First, due to a gap between the FP-CIT PET and K-NMSS assessments, we could not accurately determine the severity of NMSs in each patient at the time of each PET scan. Secondly, given that all patients in this study were medicated at the time of the K-NMSS measurements, the influence of anti-Parkinsonian medications cannot be completely excluded. Higher levodopa-equivalent doses in the HNM-PD group could contribute to certain NMSs, particularly those related to dopaminergic medications. Finally, this study was not a longitudinal follow-up study, and was thus, inadequate for the investigation of disease progression. Therefore, a further study with longitudinal follow-up is warranted to confirm our finding that the LNM-PD group shows a more benign course of motor progression. In conclusion, the present study has demonstrated that PD patients with low non-motor burden show a similar striatal dopamine depletion pattern, but may have a more benign course of motor symptom progression compared to those with high non-motor burden.

## Supporting Information

S1 Fig UPDRS-motor score and dopamine transporter (DAT) binding to the sensorimotor striatum in all patients. UPDRS-motor score was negatively correlated with DAT binding to the sensorimotor striatum (*r* = -0.314, *p* < 0.001).
(TIF)

S2 Fig Total K-NMSS score and DAT binding to the striatal sub-regions. Total K-NMSS score was not correlated with DAT binding to the limbic striatum (A), associative striatum (B) and sensorimotor striatum (C).
(TIF)
