# Supplementary figures and images for: Striatal Dopamine Depletion Patterns and Early Non-Motor Burden in Parkinsons Disease
Source: PLoS One. 2016 Aug 16;11(8):e0161316. doi: 10.1371/journal.pone.0161316 (PMC4986981; doi:10.1371/journal.pone.0161316)

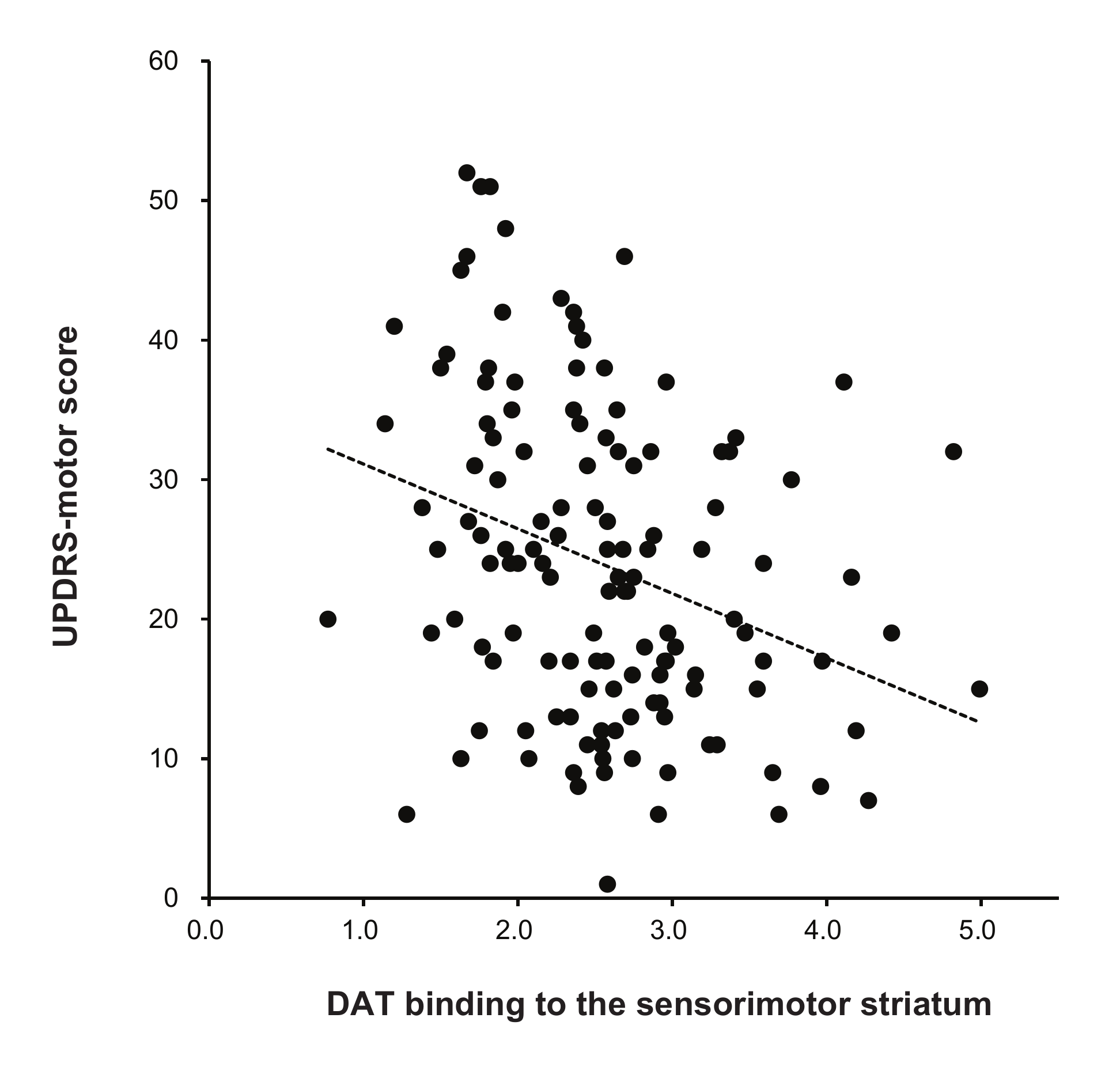

Supplement: S1 Fig — UPDRS-motor score was negatively correlated with DAT binding to the sensorimotor striatum (r = -0.314, p < 0.001). (TIF) [file pone.0161316.s001.tif]

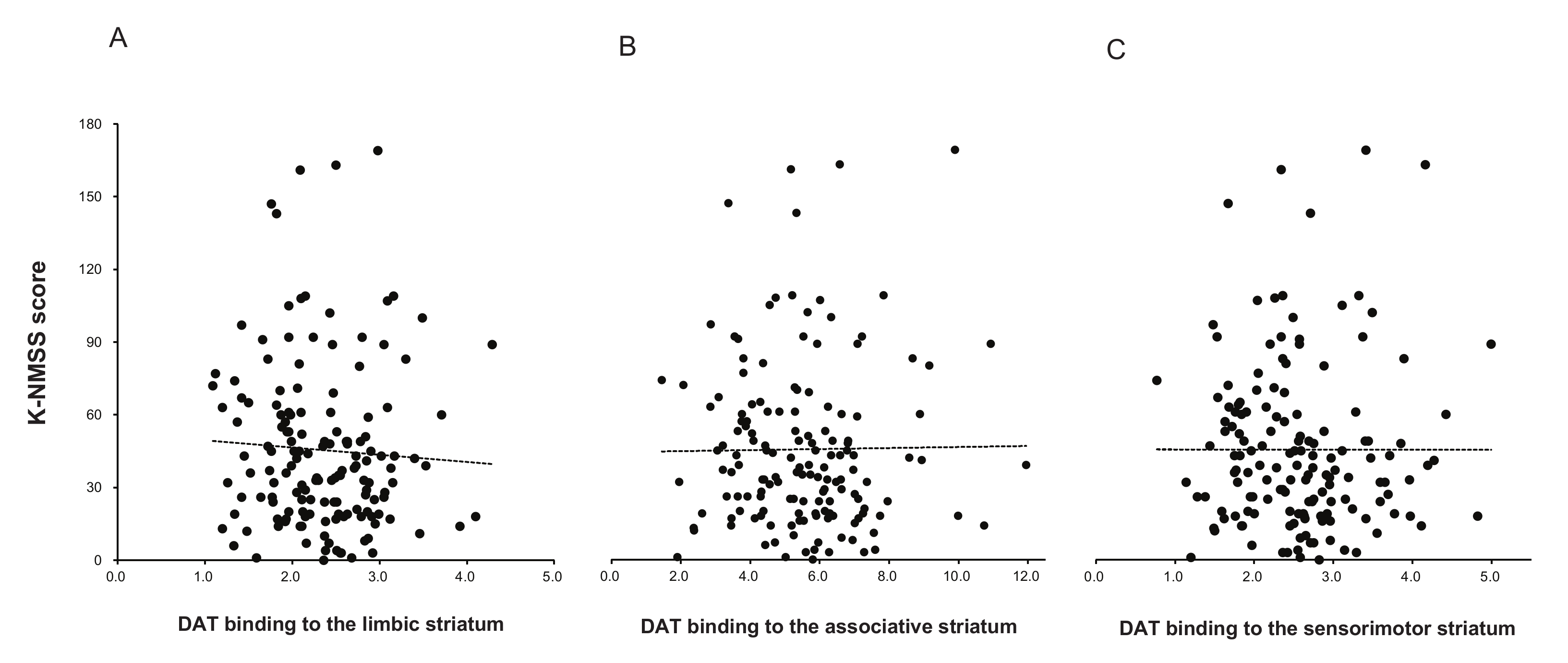

Supplement: S2 Fig — Total K-NMSS score was not correlated with DAT binding to the limbic striatum (A), associative striatum (B) and sensorimotor striatum (C). (TIF) [file pone.0161316.s002.tif]
